# Distinct von Hippel-Lindau gene and hypoxia-regulated alterations in gene and protein expression patterns of renal cell carcinoma and their effects on metabolism

**DOI:** 10.18632/oncotarget.3456

**Published:** 2015-03-27

**Authors:** Sandra Leisz, Kristin Schulz, Susanne Erb, Peter Oefner, Katja Dettmer, Dimitrios Mougiakakos, Ena Wang, Francesco M. Marincola, Franziska Stehle, Barbara Seliger

**Affiliations:** ^1^ Institute of Medical Immunology, Martin Luther University Halle-Wittenberg, 06112 Halle (Saale), Germany; ^2^ Institute of Functional Genomics, University of Regensburg, 93053 Regensburg, Germany; ^3^ Department of Internal Medicine 5, University of Erlangen, 91054 Erlangen, Germany; ^4^ Sidra Medical and Research Center, PO Box 26999, Doha, Qatar

**Keywords:** Hypoxia, von Hippel Lindau gene, renal cell carcinoma, cell metabolism, aerobic glycolysis

## Abstract

During the last decade the knowledge about the molecular mechanisms of the cellular adaption to hypoxia and the function of the “von Hippel Lindau” (VHL) protein in renal cell carcinoma (RCC) has increased, but there exists little information about the overlap and differences in gene/protein expression of both processes. Therefore the aim of this study was to dissect VHL- and hypoxia-regulated alterations in the metabolism of human RCC using ome-based strategies. The effect of the VHL- and hypoxia-regulated altered gene/protein expression pattern on the cellular metabolism was analyzed by determination of glucose uptake, lactate secretion, extracellular pH, lactate dehydrogenase activity, amino acid content and ATP levels. By employing VHL^−^/VHL^+^ RCC cells cultured under normoxic and hypoxic conditions, VHL-dependent, HIF-dependent as well as VHL-/HIF-independent alterations in the gene and protein expression patterns were identified and further validated in other RCC cell lines. The genes/proteins differentially expressed under these distinct conditions were mainly involved in the cellular metabolism, which was accompanied by an altered metabolism as well as changes in the abundance of amino acids in VHL-deficient cells. In conclusion, the study reveals similarities, but also differences in the genes and proteins controlled by VHL functionality and hypoxia thereby demonstrating differences in the metabolic switch of RCC under these conditions.

## INTRODUCTION

Renal cell carcinoma (RCC) represents approximately 2–3% of all cancers worldwide. It is a heterogeneous disease with an increasing incidence and more than 100.000 deaths per year [1]. While localized RCC can be cured by surgery, inoperable RCC is resistant to chemotherapy and radiotherapy and about 15% of patients respond to immunotherapy. Angiogenesis plays an important role in RCC development and progression as evidenced by the molecular genetics of the autosomal dominant von Hippel Lindau (VHL) syndrome and the clear cell RCC (ccRCC) subtype. Drugs targeting angiogenic factors have revolutionized the therapy of ccRCC and led to a marked survival benefit in patients with metastatic disease [2]. However, RCC patients often develop resistances to these drugs.

The ccRCC is often associated with a loss of the VHL gene function due to its deletion on chromosome 3p, loss of heterozygosity, promoter methylation or missense mutations. VHL mutations occur in approximately 50% of sporadic ccRCC, which prevent the VHL-mediated degradation of the hypoxia-inducible factor (HIF)α under normoxic conditions. This is also the case by hypoxia, which prevents binding of the prolylhydroxylase (PHD) to HIFα, thereby leading to the induction of HIF targets. More than 2% of human genes are directly or indirectly regulated by HIF [3]. These HIF targets modulate transcriptional regulation, cell proliferation, survival, apoptosis, motility, cytoskeletal structure, cell adhesion, angiogenesis and cellular metabolism [4]. In ccRCC, HIF1α is often not expressed, while HIF2α adopts its function [5]. Lack of VHL function or hypoxia cause a metabolic switch to aerobic glycolysis [6]. This is associated with a high glucose influx, a decreased gluconeogenesis and an increased lactate concentration in the tumor microenvironment, which is associated with an impaired immune recognition [7, 8] due to changes in the activity and function of T cells, NK cells and dendritic cells (DC) [9–12].

Generally, loss of VHL function or hypoxia has been postulated to cause identical alterations in gene/protein expression and function. However, this study identified differences in the VHL- and hypoxia-induced pathways by determining the mRNA and protein expression pattern of VHL^−^ RCC cell lines and their VHL transfectants in the presence and absence of hypoxia using cDNA microarrays, 2DE-based proteomics, and metabolomics. Selected differentially expressed genes and proteins were further validated and subjected to functional analysis.

## RESULTS

### Characterization of the different VHL model systems

**Figure 1:**
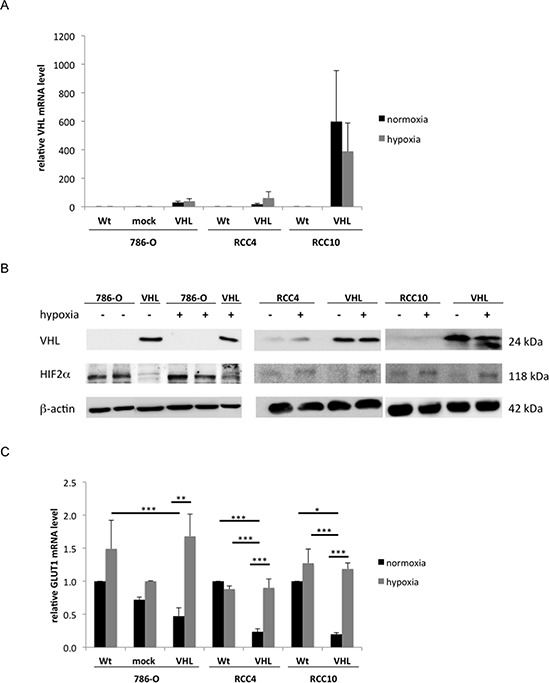
Restoration of functional VHL expression in 786-O, RCC4 and RCC10 cells **(A)** VHL^−^ cells were stably transfected with a VHL overexpression vector or vector backbone. The bulk cultures were analysed for VHL mRNA expression after 24 h incubation during normoxia or hypoxia (1% O_2_) conditions using qPCR. The data was normalized to β-actin expression and the expression of wild type cells incubated under normoxia was set to 1. **(B)** Western blot analysis of VHL and HIF2α protein in wt 786-O, RCC4, and RCC10 cells and their respective transfectants during normoxia and hypoxia. Immunostaining was performed with a VHL- and HIF2α-specific antibody as described in the experimental procedures. Equal amount of protein loading was controlled by immunostaining using β-actin-specific antibody. A representative result of three independent biological replicates is shown. **(C)** mRNA expression of HIF2α target gene GLUT1 in VHL^−^ and VHL^+^ cells during normoxia and hypoxia oxygen conditions was measured by qPCR and normalized to β-actin. The expression level of wt RCC cells was set to 1.

The mRNA and protein expression levels of VHL were determined under both normoxic and hypoxic conditions in the VHL^−^ cell lines 786-O, RCC4 and RCC10 and their VHL transfectants using qPCR (Figure 1A) and Western blot analysis (Figure 1B), respectively. Expression of VHL protein was only detectable in VHL transfectants and did not significantly differ between normoxia and hypoxia. Over-expression of VHL led to a degradation of HIF2α protein during normoxia and to its stabilization under hypoxia (Figure 1B). Subsequently, transcription levels of the HIF2α target gene GLUT 1 were reduced by 2.1 to 5-fold under normoxia in the VHL^+^ RCC cells when compared to VHL^−^ RCC cells. In contrast, hypoxia caused a 3.5 to 5.9-fold up-regulation of GLUT1 expression in VHL^+^ RCC cells when compared to normoxia (Figure 1C).

### Identification of VHL- and hypoxia-induced genes

The gene expression pattern of VHL^−^ and VHL^+^ 786-O cells was determined under normoxic and hypoxic conditions. The levels of gene expression significantly differed between the cell systems and culture conditions used (Table S2). Some genes were down-regulated up to approximately 60-fold (NID2), while others were up-regulated to approximately 25-fold (RAB31) depending on the conditions analysed.

The most differentially expressed transcripts with *n* = 1202 and *n* = 1292, respectively, were found upon comparison of VHL^+^ with VHL^−^ cells under normoxia and hypoxia (Table S3). The overlapping genes (662 genes, Figure S1, Table S4) of these groups represent putative VHL-regulated genes.

**Figure 2:**
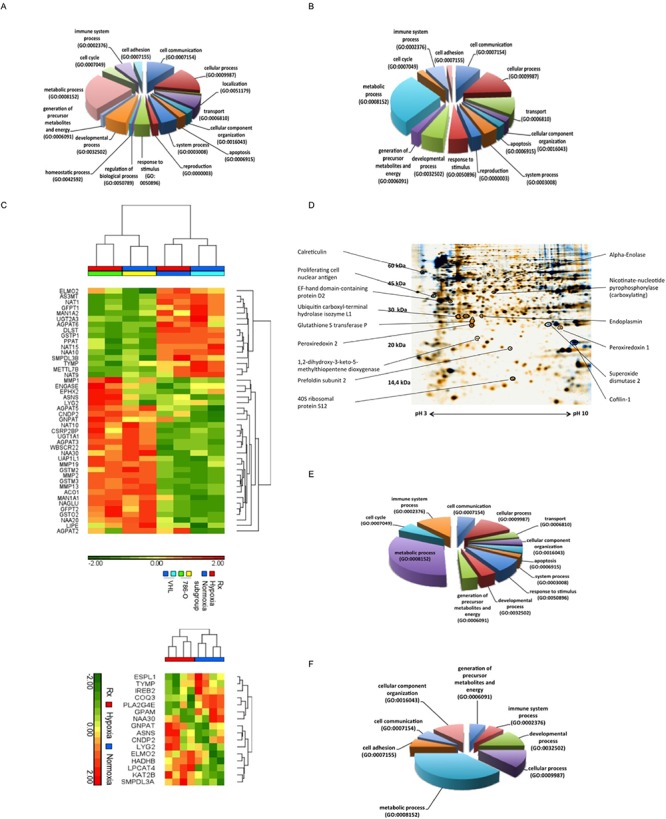
Functional classification of VHL- and hypoxia-dependent regulated genes and proteins **(A and B)** The cDNA microarray analysis was performed with Trizol-prepared total RNA as described in experimental procedures. For the classification of data in biological processes the PANTHER classification system according to gene ontology criteria was employed. The pie chart shows representative classifications for (A) VHL^+^ vs. VHL^−^ 786-O cells, and (B) wt cells incubated during hypoxia (1% O_2_) vs. normoxia. **(C)** Hierarchical clustering of “ome”-based data. Using the hierarchical clustering differentially expressed genes associated with metabolic function (*p* < 0.05) identified by one way ANOVA among VHL^+^, VHL^−^ 786-O under hypoxia or normoxia (top heat map) and hypoxia- vs. normoxia-specific metabolic function related genes independent of VHL status (*t* test, *p* < 0.05, bottom heat map) were visualized. **(D)** Identified differentially expressed proteins were marked on a representative 2-DE gel of VHL^−^ vs. VHL^+^ 786-O cells during hypoxia. **(E and F)** The pie charts demonstrate the classification of differentially expressed proteins, which were identified by 2DE followed by peptide mass fingerprint. The classification of proteins was performed with PANTHER software and is representatively demonstrated for E) VHL^+^ vs. VHL^−^ 786-O cells and F) wt cells incubated under normoxic and hypoxic (1% O_2_) conditions.

The greatest proportion of differentially expressed genes mediated by VHL expression (24%; Figure 2A) and hypoxia (28%; Figure 2B) exert a metabolic function. Figure 2C summarizes the hierarchical clustering of the differentially expressed metabolic genes in VHL-deficient vs. VHL-expressing cells under normoxia and/or hypoxia (*p* < 0.05). The number of VHL-independent, hypoxia-regulated genes (194 regulated cDNAs) was much lower (Table S2), but the lowest number was found for putative HIF2α–regulated genes (28 genes, Table S4). These data indicated distinct VHL-, hypoxia- as well as HIF-independent regulated processes.

### Identification of VHL- and hypoxia-regulated targets

In order to identify differentially expressed proteins caused by a distinct VHL status and hypoxic environment, 2DE-based proteomics of VHL^−^ and VHL^+^ 786-O cells cultured under normoxia or hypoxia was performed leading to 76 differentially expressed proteins identified by MALDI-TOF/MS analysis (Table S3, Table S5). These included VHL-independent, but hypoxia-dependent as well as VHL-dependent and hypoxia-independent and HIF2α-dependent targets, respectively. Figure 2D shows identified differentially expressed proteins on a representative 2-DE gel of VHL^−^ vs. VHL^+^ 786-O cells during hypoxia. Although the differentially expressed proteins were associated with distinct functions, approximately 30% of VHL-regulated proteins and 38% of hypoxia-regulated proteins belong to metabolic processes (Figure 2E, 2F). There exists a significant overlap in differentially expressed proteins by comparing VHL^−^ vs. VHL^+^ 786-O protein profiles to that of 786-O cells under normoxic versus hypoxic conditions, whereas only a few proteins were found to be differentially expressed in normoxic vs. hypoxic 786-O cells (*n* = 13) and VHL^+^ 786-O cells (*n* = 2), respectively (Table S5).

### Impact of VHL- and hypoxia-dependent alterations on the cellular metabolism

In order to validate the differentially expressed genes and proteins, modulated by VHL, hypoxia or a combination of both, qPCR, Western blot analyses and enzymatic activity assays of selected targets were performed.

**Figure 3:**
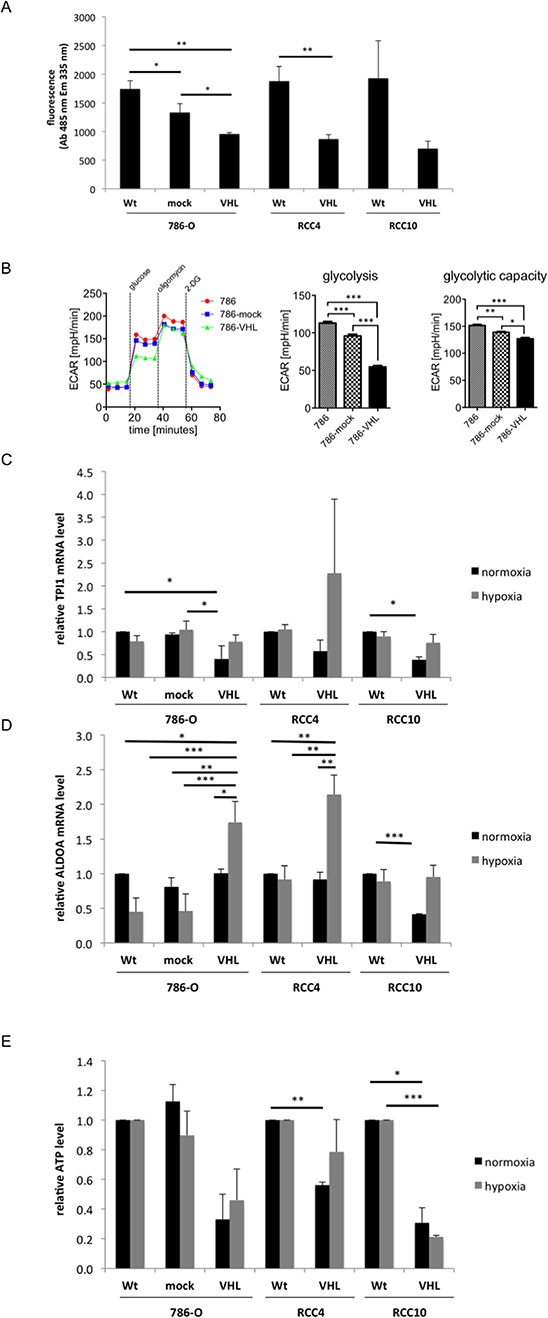
VHL-dependent changes in glucose uptake and glycolysis **(A)** 1 × 10^4^ cells/cell line were seeded and the glucose uptake was measured by incorporation of fluorescently labelled deoxyglucose (2-NBDG) at absorption and emission wavelengths of 485 nm and 535 nm, respectively. The fluorescence signal of wild type (wt) cells was set to 1. The figure shows the mean values and standard derivations of three independent assays. **(B)** The extracellular acidification rate of wt, mock- and VHL-transfected 786-O cells is measured in response to the indicated substrates and inhibitors. ECAR is predominantly the result of anaerobic glycolysis. ECAR levels upon administration of glucose are indicative for basal glycolysis and upon administration of oligomycin for the cells' glycolytic capacity. A representative analysis as well as the mean values for glycolysis and the glycolytic capacity is shown. Bars indicate the standard error mean. Abbreviations: p, *p*-value; *, *p* < 0.05; **, *p* < 0.005; ***, *p* < 0.001. **(C and D)** Analyses of TPI1 (C) and ALDOA (D) mRNA expression are shown. qPCR analyses of wt and VHL-transfected 786-O, RCC4 and RCC10 cells were performed using oligo-dT primed cDNA and normalized to β-actin mRNA expression. The expression levels of parental cells were set to 1 and the diagram shows the means with S.D. of three biological replicates. **(E)** ATP levels were measured by the Cell Titer Glo luminescence cell viability assay as described in experimental procedures. The VHL^−^ RCC cells were set to 1.

The glucose consumption of the VHL^−/+^ RCC model system was investigated via the uptake of fluorescent dye labelled glucose. The VHL^+^ RCC cells showed a statistically significant ≅2-fold reduced glucose uptake compared to the VHL-deficient RCC cells (Figure 3A), which was accompanied by an altered expression of many glycolytic enzymes (Table S6). In addition, extracellular flux analysis was performed to assess the ECAR. As shown in Figure 3B, VHL expression significantly decreased glycolysis and glycolytic activity. A VHL-dependent down-regulation of pyruvate kinase (PK)M2, γ-enolase (ENO2) and triosephosphate isomerase (TPI)1 was detected, while ENO2, TPI1 and aldolase (ALDO)A were hypoxia-dependently up-regulated (Table S6). Over-expression of VHL caused a 2- to 2.5-fold reduction in the expression of TPI1 under normoxic conditions in comparison to VHL-deficient RCC cells, while its expression was enhanced 2- to 3.9-fold under hypoxia in the VHL^+^ RCC cells when compared to normoxic conditions (Figure 3C). In VHL^−^ 786-O and RCC4 cells, the expression of ALDOA is hypoxia-dependent 1.7- to 2-fold up-regulated, while in RCC10 cells a 2.5-fold VHL-mediated down-regulation of ALDOA was observed, which could be restored by hypoxia (Figure 3D). Furthermore, the intracellular ATP levels were up to 67% decreased in VHL^+^ when compared to VHL^−^ RCC cells (Figure 3E).

### Association of VHL expression with an induction of the citric acid cycle and the mitochondrial respiratory chain

**Figure 4:**
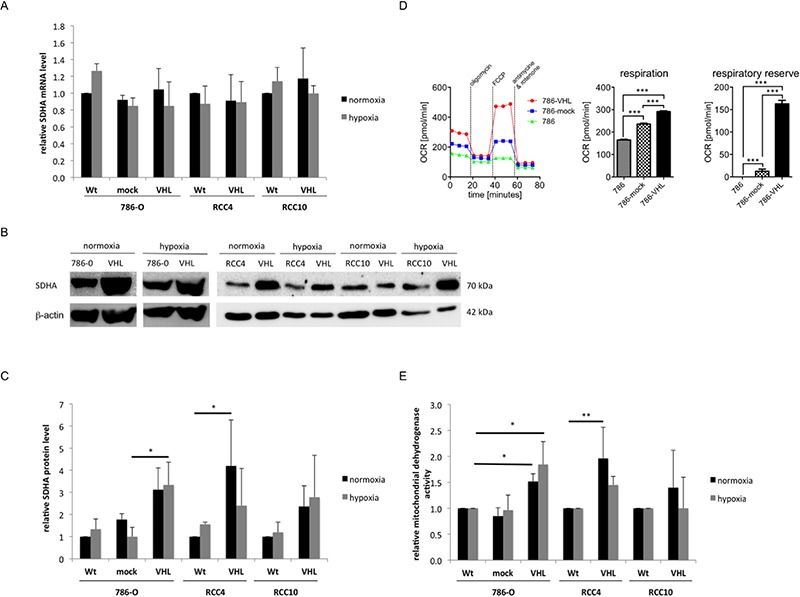
Induction of TCA and mitochondrial respiratory chain through VHL overexpression **(A)** The transcription level of SDHA was determined using oligo-dT primed cDNA and specific primer via qPCR. Expression of wt cells was set to 1. **(B)** 50 μg protein/cell line was separated by 10% SDS-PAGE, transferred onto a nitrocellulose membrane before immunostaining was performed with a SDHA-specific antibody as described in experimental procedures. Equal protein loading was controlled by subsequent immunostaining using an anti-β-actin-specific antibody. A representative result of three independent biological replicates is shown. **(C)** Densitometric analysis of SDHA expression in VHL^−^ and VHL^+^ 786-O, RCC4 and RCC10 cells incubated during normoxia and hypoxia (1% O_2_) is shown. Areas of immunoblot signals were integrated using AIDA image analyser software and normalized to β-actin. The graphic showed the means and S.D. of three independent experiments. **(D)** The baseline oxygen consumption rate (OCR) was measured by an XFe96 flux analyzer in wt, mock-, and VHL-transfected 786-O cells. OCR is an indicator of mitochondrial respiration. Respiration (OCR) is measured under basal conditions and in response to the indicated substances. Changes after application of the uncoupling agent FCCP are indicative for the respiratory reserve. A representative analysis as well as the mean values for basal respiration and the respiratory reserve is shown. Bars indicate the standard error mean. Abbreviations: p, *p*-value; ***, *p* < 0.001. **(E)** 5 × 10^3^ cells/well were seeded in triplicates in 96-well plates and incubated for 72 h at 37°C and 5% CO_2_ during normoxia and hypoxia. The activity of mitochondrial dehydrogenases was measured trough the turnover of the tetrazolium salt XTT to formazan by the OD of 490 nm.

To further analyse the VHL status-dependent metabolic changes, the tricarboxylic acid cycle (TCA) and the mitochondrial respiratory chain activity was determined in VHL^−^/VHL^+^ model systems. Despite a VHL-dependent suppression of glycolysis and ATP production, the expression of the enzymes of the TCA and, consequently, of the mitochondrial respiratory chain were increased (Table S6). Interestingly, the SDHA transcription was neither dependent on VHL nor on hypoxia (Figure 4A). In contrast, the protein expression of SDHA was approximately 2-fold up-regulated in VHL^+^ RCC cells (Figure 4B, 4C). The mitochondrial respiration was assessed in VHL^−^/VHL^+^ RCC cells using the extracellular flux assay. An increase of basal oxygen associated with an increased respiration and respiratory reserve was found in VHL^+^ compared to VHL^−^ RCC cells (Figure 4D). This was associated with a 1.5 to 2-fold increase of the mitochondrial dehydrogenase activity upon VHL overexpression (Figure 4E).

### VHL-dependent regulation of lactate secretion and pH regulation

**Figure 5:**
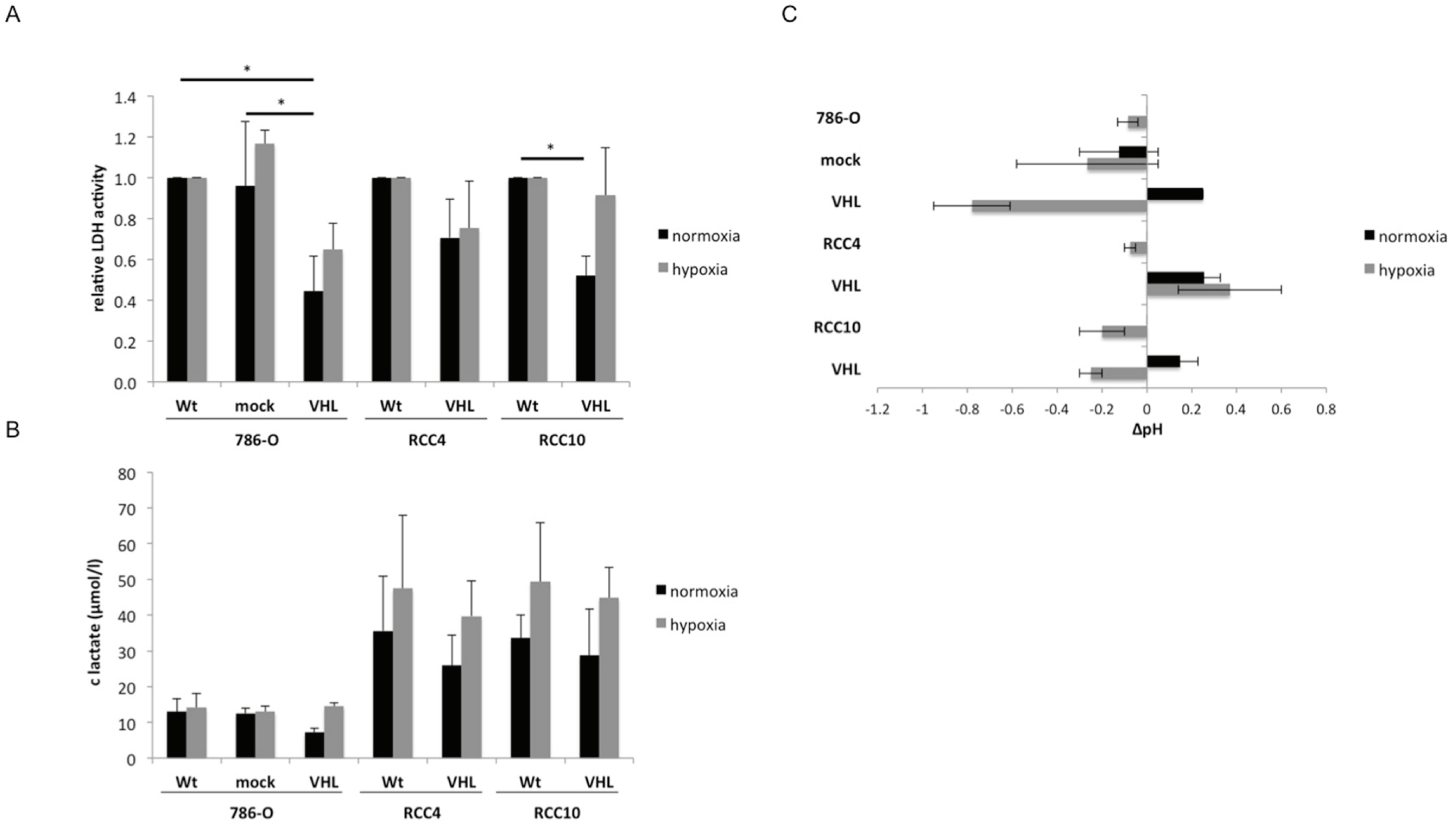
VHL-dependent alterations of lactate dehydrogenase activity, lactate secretion and extracellular pH as a function of oxygen tension Five × 10^3^ VHL^−^/VHL^+^ cells were seeded in triplicate in 96-well plates and incubated for 48 h under both normoxic and hypoxic conditions. Shown are the means and standard derivations of three independent assays **(A)** The intracellular lactate dehydrogenase activity was determined by the CytoTox96 Non-Radioactive Cytotoxicity Assay (Promega). The LDH activity of wt RCC cells was set to 1. **(B)** Extracellular lactate levels in the cell culture supernatants of VHL^−^ and VHL^+^ RCC cells under both normoxic and hypoxic conditions. **(C)** Changes in the pH value of supernatants in relation to the respective VHL-deficient RCC cell line under normoxia.

Since VHL could regulate the lactate dehydrogenase (LDH) expression [18, 19], the intracellular LDH activity of the VHL^−^ and VHL^+^ cells was determined. When compared to VHL^−^ RCC cells, LDH activity was down-regulated between 30% to 66% in VHL transfectants (Figure 5A), which was associated with an up to 55% lower extracellular lactate concentration (Figure 5B) and an altered pH in the cell culture supernatant (Figure 5C). In contrast, hypoxia restored the VHL-mediated reduction of LDH activity and lactate secretion, which is accompanied by a drop in the extracellular pH of the VHL transfectants (Figure 5).

### Altered VHL-dependent amino acid content in RCC cell lines

**Figure 6:**
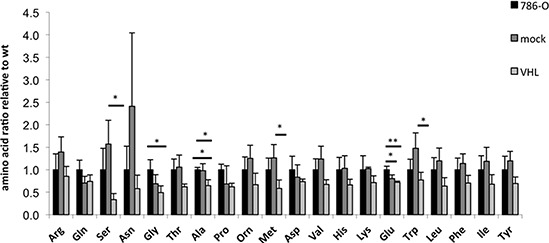
Intracellular free amino acid levels of wt, VHL^+^ and VHL^−^ 786-O cells Amino acid concentrations are expressed as ratios relative to the wt, which was set to one. A one-way ANOVA followed by Tukey post hoc test was performed to test for significant differences (*p ≤ 0.05, **p ≤ 0.01).

In order to investigate whether the amino acid metabolism is also altered by the VHL status, the content of 20 free amino acids was measured in the 786-O VHL model system under normoxic conditions. In comparison to VHL-deficient 786-O cells, the overall content of free amino acids was reduced about 25% in VHL^+^ RCC cells, with the highest down-regulation of 2.5-fold for serine (Figure 6). In contrast, the arginine concentration was not altered in the presence or absence of VHL.

## DISCUSSION

Major advances have been made in our understanding of the molecular mechanisms of cellular adaption to hypoxia and the function of VHL. Nevertheless, little information exists about the overlap and differences of VHL-dependent/independent and hypoxia-dependent alterations in gene and protein expression patterns and their effects on the cellular metabolism. Therefore, the aim of the study was to dissect both the VHL- and/or hypoxia-induced pathways, which might lead to the identification of candidate biomarkers for disease progression and novel therapeutic targets. Using a tandem approach of cDNA microarrays and 2-DE-based proteome analyses, a large number of differentially expressed genes and/or proteins could be detected in VHL^+^ versus VHL^−^ RCC cells as well as under normoxic and/or hypoxic conditions. Despite a high frequency of genes/proteins concordantly regulated by loss of VHL function and hypoxia, a number of differentially expressed genes/proteins demonstrating a VHL-dependent, VHL-independent, hypoxia-dependent, and hypoxia-independent regulation, respectively, of the gene and protein expression profiles was identified. qPCR and/or Western blot validation further confirmed the differential gene and protein expression profiles in the VHL^−^ and VHL^+^ RCC model systems analysed in the absence or presence of hypoxia. Reflecting the biology and molecular features of RCC, the differentially expressed genes/proteins were mainly involved in the cell metabolism and were localized in the cytoplasm. In most cases there exists a concordant expression of these molecules at the mRNA and protein level, although some genes appear to be post-transcriptionally controlled, such as SDHA. These data are in line with the proteome analysis of primary RCC lesions and patient-matched normal kidney epithelium demonstrating an altered glycolysis and gluconeogenesis [20, 21]. This study here extended these results by analysing the VHL and hypoxia dependence or independence demonstrating a link between VHL loss and hypoxia, but also a distinction between both pathways. This is mediated by an altered gene and protein expression pattern, which also modulates the tumor microenvironment thereby differentially affecting the metabolic switch of RCC.

Heterogeneous, but increased expression of ALDOA was found in RCC subtypes when compared to normal kidney cortex [22] as well as in the serum of RCC patients and have been used for tumor staging [23]. These data were extended by demonstrated an up-regulation of ALDOA expression associated with hypoxic conditions and loss of VHL expression suggesting a link between ALDOA, VHL and hypoxia. Thus, ALDOA might represent a suitable and potential target irrespective of the VHL status and oxygen conditions.

Several reports have demonstrated a direct correlation between the loss of VHL expression and alterations in the cellular metabolism, leading to an altered tumor microenvironment, such as high lactate concentrations and low pH value [24]. This was also confirmed in this study by reversible hypoxia- and VHL loss-mediated changes of the intracellular lactate dehydrogenase levels, extracellular lactate concentrations and extracellular pH, as well as the differential expression of genes/proteins involved in changes of the tumor microenvironment. These alterations could lead to an immune suppressive tumor microenvironment [25] although a correlation of the VHL status with the immune cell infiltration has not yet been determined in detail.

Metabolome analysis demonstrated an altered metabolite and amino acid content in VHL^−^ vs. VHL^+^ cells (Figure S2), thus supporting their distinct metabolism and was further strengthened by recent work of Gameiro and co-authors describing reduced intracellular citrate levels in VHL mutant versus VHL wt RCC cells due to the presence of HIF thereby linking VHL and hypoxia and sensitizing VHL-deficient cells to glutamine deprivation [26]. Citrate levels could be restored by silencing PDK-1 and ACLY, which was accompanied by suppression of RCC. Furthermore, HIF rendered VHL-deficient cells sensitive to glutamine deprivation *in vitro*, while systemic treatment with glutaminase inhibitors suppressed the *in vitro* and *in vivo* growth of RCC cells.

## MATERIALS AND METHODS

### Cell lines and hypoxia incubation

The RCC cell lines RCC4 and RCC4 VHL were obtained from Health Protection Agency Culture Collections (Salisbury, UK). The RCC cell lines 786-O and RCC10, and their respective stable VHL transfectants were kindly provided by Prof. Wiesener (Erlangen) and were authenticated by DNA profiling. All cell lines were maintained in DMEM supplemented with 10% fetal calf serum (Gibco), 2 mM glutamine (Biochrom AG), 1 mM pyruvate (Gibco), 100 U/mL penicillin and 100 μg/mL streptomycin (PAA). Cells were cultivated for 24 to 72 h either under normal oxygen conditions or at 1% oxygen (hypoxia incubator, Binder) at 37°C and 5% CO_2_. All experiments were carried out during the logarithmic growth phase of the cells.

### Microarray analysis

Total RNA was extracted from 3 × 10^6^ cells using Trizol reagent (Invitrogen), reverse transcribed and amplified with the WT expression kit (Invitrogen) according to the manufacturer's manual. Fragmented cDNA was end-labeled using the Affymetrix terminal labeling kit and hybridized onto Human GeneChip 1.0 ST arrays (Affymetrix). The chips were stained and washed following standard procedure and scanned on a GeneChip Scanner 3000 7G (Affymetrix).

### Two-dimensional gel electrophoresis

Protein was isolated from 5 × 10^7^ VHL^−^/VHL^+^ 786-O cells incubated for 48 h under normoxic or hypoxic conditions. 550 μg/protein lysate were loaded onto IPG strips (pH 3–10, non-linear, Amersham Biosciences) followed by an isoelectric focusing and second-dimension SDS-PAGE separation (13%) and staining with colloidal Coomassie as described [13]. The gels were analyzed using the Delta2D software package (Decodon). Proteins found to be at least two-fold regulated (factor ≥ 2.0 or ≤ 0.50; *p* ≤ 0.05) were subjected to mass spectrometric identification. The two-dimensional gel electrophoresis was carried out in three independent experiments using five gels for each condition.

### In-gel digestion and mass spectrometric analysis

Differentially regulated protein spots were excised from the gels using the Spothunter (Herolab), destained with 50% acetonitrile (v/v) over night and subjected to in-gel digestion with 8 μg/mL porcine trypsin (Promega). Tryptic digests were subjected to MALDI-TOF mass spectrometry (Ultraflex, Bruker). Protein identification was performed as previously described [13].

### RNA isolation and real time quantitative PCR

Total cellular RNA was isolated using the NucleoSpin RNA II kit (Macherey & Nagel). Two μg of total RNA were reverse transcribed into cDNA using the Revert H Minus First Strand cDNA synthesis kit (Fermentas) and oligo(dT)18 primer according to the manufacturer's instructions. Comparative quantification of gene expression was performed as previously described [14]. The target-specific primers used for qPCR are listed in the Supplementary Table S1.

### Western blot analysis

Cells were harvested as previously described [13] and protein isolated according to Laemmli [15]. Fifty μg protein/lane were subjected to Western blot analysis as recently described [13]. Membranes were incubated over night at 4°C with the primary monoclonal antibodies (mAb) directed against succinate dehydrogenase complex subunit A (SDHA; Cell Signaling), HIF2α (Novus) and β-actin (Sigma-Aldrich), followed by incubation for 1 h with horseradish peroxidase-linked secondary antibody and developed using the ECL method. Chemoluminescence signals were detected using a CCD camera (LAS3000, Raytest). For quantification of the SDHA protein expression the respective area of the signal was integrated using an AIDA image analyser (Raytest) and subsequently normalized to β-actin.

### Measurement of lactate and mitochondrial dehydrogenase activity

5 × 10^3^ VHL^−^/VHL^+^ cells were seeded in triplicates in 96-well plates and incubated for 48 h under normoxic or hypoxic conditions. The intracellular lactate dehydrogenase activity was determined using the CytoTox96 Non-Radioactive Cytotoxicity Assay (Promega) according to the manufacturer's instructions. Mitochondrial dehydrogenase activity was measured by the conversion of the monotetrazolium salt XTT to formazan as previously described [16]. The experiments were carried out in three independent assays and the normalization was performed against the parental cell line.

### Determination of lactate concentration and extracellular pH

Lactate concentrations were measured using the lactate detection kit (Sigma) according to the manufacturer's instructions using supernatants of VHL^−^ and VHL^+^ cells incubated for 48 h under normoxic and hypoxic conditions. In parallel, the extracellular pH was determined in the same supernatants used using a digital pH meter (p525, WTW). All experiments were carried out in three independent assays and the results are represented as the mean value of assays.

### Measurement of intracellular ATP and glucose uptake

For determination of the intracellular ATP content 5 × 10^3^ cells were seeded in 96-well plates and incubated for 24 h under normoxia or hypoxia. The intracellular ATP levels were determined using the Cell Titer Glo luminescence cell viability assay (Promega) according to the manufacturer's instructions. Luminescence was measured using the Lumat LB 9507 luminometer (Berthold Technologies). Values were normalized to wild type cells cultured under normoxic conditions.

The glucose uptake was measured with fluorescence labelled 2-deoxy-2-[(7-nitro-2, 1, 3-benzoxadiazol-4-yl)amino]-D-glucose (2-NBDG, Cayman Biochemical). Briefly, 1 × 10^4^ cells were cultured for 24 h in 96-well black/clear tissue culture imaging plates (BD). Cells were washed twice with PBS, treated with 0.1 mM 2-NBDG in 50 μL PBS for 20 min and washed again. The fluorescence signal was determined using a Tecan Infinite M200 fluorescence reader (absorption 485 nm, emission 535 nm).

### Extracellular flux assay

2.5 × 10^5^ cells/well were seeded in 96XF micro plate (Seahorse Bioscience) and incubated over night at 37°C and 5% CO_2_. One hour prior to measurement with the XF96e Extracellular Flux Analyzer (Seahorse Bioscience) cells were incubated at 37°C in a CO_2_-free atmosphere. First, the basal oxygen consumption rate (OCR) as indicator for mitochondrial respiration and extracellular acidification rate (ECAR) as indicator for glycolysis were detected. Next, OCR and ECAR were determined after the addition of 1 mM oligomycin, 2.5 mM FCCP (Carbonyl cyanide-p-trifluoromethoxy-phenylhydrazone) and a combination of 3 mM antimycin and 3 mM rotenone (XF Cell Mito Stress Test Kit, Seahorse Bioscience) as well as 10 mM glucose, 1 mM oligomycin and 100 mM 2-DG (XF Cell Glyco Stress Test Kit, Seahorse Bioscience). Experiments were performed in octaplicates.

### Amino acid analysis

2.5 × 10^6^ cells were seeded in a 6-well plate for 24 h before the supernatant was collected. The cells were washed twice with PBS, scraped in ice-cold 80% aqueous methanol and directly frozen at –80°C until use. The sample was centrifuged at 4°C and 5.725 × g for 5 min and the extract was collected. The protein pellet was then washed twice with 80% aqueous methanol, the extracts were combined and dried in a vacuum evaporator (CombiDancer, Hettich AG). The residue was reconstituted in 100 μL water. Amino acid analysis was performed by HPLC-ESI-MS/MS using 10 μL cell extract following a propyl chloroformate derivatization protocol as recently described [17].

### Statistical analysis

Microarray: Data analyses were performed using Partek Genomic Suite (Partek) software after Robust Multichip Average (RMA) normalization. Differentially expressed genes were defined by one-way ANOVA with a *p* value < 0.005 and for included metabolic genes of hierarchical clustering *p* < 0.05.

qPCR and metabolic assays: Statistical analysis was performed with one-way ANOVA followed by Tukey post hoc test or unpaired Student's *t* test. Significance was accepted if *p* values were ≤ 0.05 (*p ≤ 0.05, **p ≤ 0.01, ***p ≤ 0.001). Data were expressed as the mean ± S.D.

### Data integration and network analysis

Differentially expressed genes were further functionally analyzed using Ingenuity Pathway Analysis (IPA, Ingenuity) and Gene Ontology enrichment analysis integrated in the Partek software.

## SUPPLEMENTARY FIGURES AND TABLES







## Abbreviations

2-NBDG: 2-[N-(7-nitrobenz-2-oxa-1, 3-diazol-4-yl)amino]-2-deoxy-D-glucose
2DE: two-dimensional gel electrophoresis
ACO: aconitase
ccRCC: clear cell renal cell carcinoma
ALDOA: aldolase A
AKR1B1: aldose reductase
DMEM: Dulbecco's Modified Eagle's Medium
ECAR: extracellular acidification rate
ECL: electrochemiluminescence
ECHS1: enoyl-CoA hydratase short chain 1
ENO: enolase
FCS: fetal calf serum
GLUT: glucose transporter
HIF: hypoxia inducible factor
HRE: hypoxia responsible element
LDH: lactate dehydrogenase
MALDI-TOF: matrix-assisted laser desorption/ionization time-of-flight
MAPK: mitogen-activated protein kinase
MCT: monocarboxylate transporter
OCR: oxygen consumption rate
p: *p*-value
PBS: phosphate-buffered saline
PKM: pyruvate kinase
RCC: renal cell carcinoma
SDH: succinate dehydrogenase
SDHA: succinate dehydrogenase complex subunit A
TCA: tricarboxylic acid cycle
TPI: triose-phosphate isomerase
VHL: von Hippel-Lindau
Wt: wild type
