# Supplementary material for: Distinct von Hippel-Lindau gene and hypoxia-regulated alterations in gene and protein expression patterns of renal cell carcinoma and their effects on metabolism
Source: Oncotarget. 2015 Mar 27;6(13):11395–406. doi: 10.18632/oncotarget.3456 (PMC4484464; doi:10.18632/oncotarget.3456)
Supplement: Supplementary file 1 [file oncotarget-06-11395-s001.pdf]

# SUPPLEMENTARY FIGURES AND TABLES

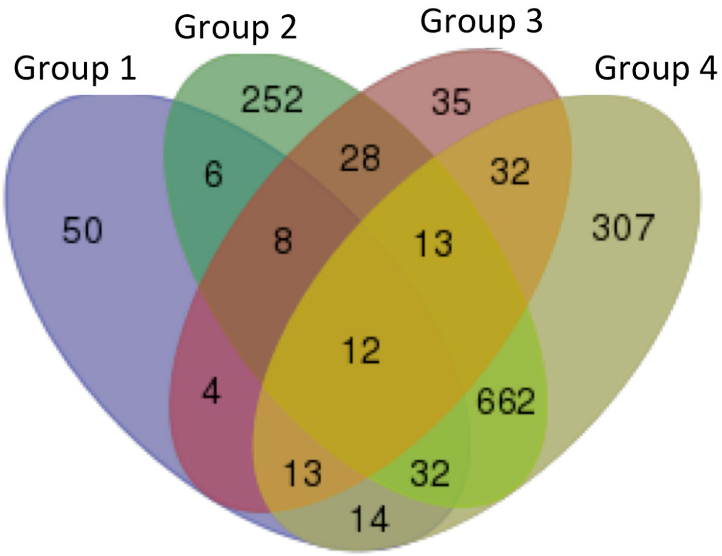


**Supplementary Figure S1: Number of overlapping genes of the comparison of 786-O VHL$^-$ and VHL$^+$ cells during normoxia and hypoxia.** The venn diagram shows the overlapping differentially expressed genes identified via cDNA microarray as described in materials and methods. Group 1: 786-O hypoxia vs. 786-O normoxia; group 2: VHL normoxia vs. 786-O normoxia; group 3: VHL hypoxia vs. VHL normoxia; group 4: VHL hypoxia vs. 786-O normoxia;

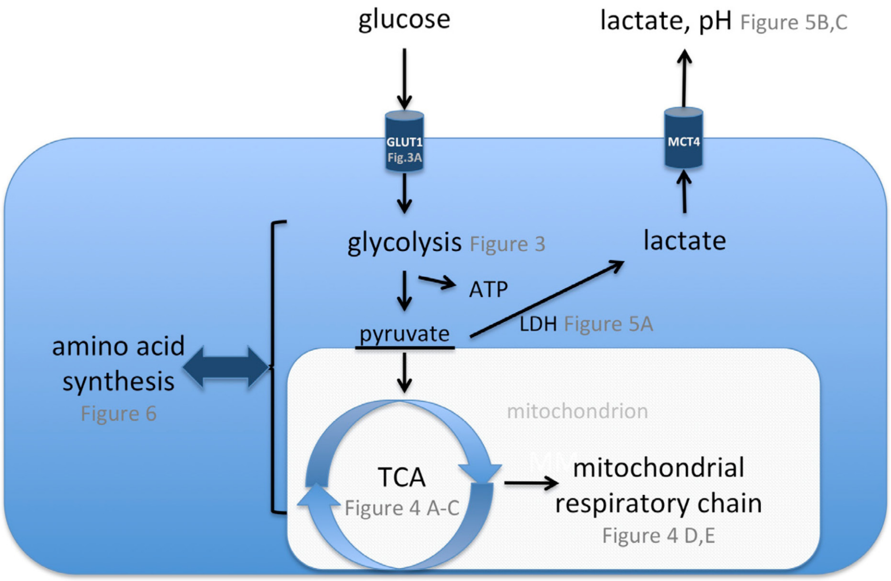


**Supplementary Figure S2: Altered metabolism in VHL-deficient RCC cells.** The scheme presents a summary of the metabolic changes observed in VHL-deficient RCC cells. Changes in glucose uptake, expression of glycolysis and TCA enzymes, intracellular ATP production, amino acid content, activity of mitochondrial dehydrogenase and lactate dehydrogenase, lactate secretion, and extracellular pH were measured in three independent RCC VHL cell models.

**Supplementary Table S1: Primer sequences for qPCR**

| Primer name | Primer sequence | Reference sequence |
|---|---|---|
| VEGFAfwd | AACCATGAACTTTCTGCTGTCTTGG | NM_001171630 |
| VEGFArev | ATCAGGGTACTCCTGGAAGATGTCC | |
| GLUT1fwd | CTTCACTGTCGTGTCGCTGTTTGT | NM_006516 |
| GLUT1rev | AAATTTGAGGTCCAGTTGGAGAAGC | |
| ACTBfwd | GGACTTCGAGCAAGAGATGG | NM_001101 |
| ACTBrev | AGCACTGTGTTGGCGTACAG | |
| VHLfwd | CATCCGTTGATGTGCAATG | NM_198156 |
| VHLrev | GAAAGAGCGATGCCTCCA | |
| SDHAfwd | GATTACTCCAAGCCCATCCA | NM_004168 |
| SDHArev | CACAGTCAGCCTCGTTCAAA | |
| ECHS1fwd | CAGTCATCGCTGCTGTCAAT | NM_004092 |
| ECHS1rev | AGTGAGGACCATCTCCATCG | |
| ACO1fwd | CTTTCCTGCTGGGAATCAAA | NM_002197 |
| ACO1rev | TCCAGCTTGACCTGGACTTT | |
| FAHfwd | CGAGCCCTACACATTTGACA | NM_000137 |
| FAHrev | CATGGAGCCGAAGTTTTCTG | |
| ALDOAfwd | GTGCTGGCTGCTGTCTACAA | NM_000034 |
| ALDOArev | TCCAGACAGGAAGGTGATCC | |
| TPI1fwd | CCCTGGCATGATCAAAGACT | NM_000365 |
| TPI1rev | TCTGCGATGACCTTTGTCTG | |

**Supplementary Table S2: cDNA microarray data of 786-O VHL$^-$ and VHL$^+$ RCC cells incubated under different oxygen conditions**

**Supplementary Table S3: Number of VHL- and/or hypoxia-regulated genes and proteins**

| sample | 786-O hypoxia vs. 786-O normoxia | VHL normoxia vs. 786-O normoxia | VHL hypoxia vs. VHL normoxia | VHL hypoxia vs. 786-O normoxia |
|---|---|---|---|---|
| microarray total number | 194 | 1202 | 186 | 1292 |
| Up-regelated | 106 | 538 | 102 | 606 |
| Down-regulated | 88 | 664 | 84 | 686 |
| 2DE total number | 9 | 28 | 2 | 39 |
| Up-regulated | 9 | 15 | 2 | 28 |
| Down-regulated | 0 | 13 | 0 | 11 |

Differentially expressed cDNAs and proteins of the comparison of VHL$^-$/VHL$^+$ 786-O cells during normoxia and hypoxia incubation. Transcriptome and proteome analysis were determined as outlined in materials and methods. Differentially expressed genes were defined by one-way ANOVA with a $p$ value <0.005 and proteins found to be at least two-fold regulated (factor $\geq$ 2.0 or $\leq$ 0.50; $p \leq 0.05$).

**Supplementary Table S4: Overlap of different expressed genes of the comparison of 786-O VHL$^-$ and VHL$^+$ cells during normoxia and hypoxia**

**Supplementary Table S5: Differentially expressed proteins identified by 2DE followed by peptide mass fingerprint**

| Setting | Gene Symbol | Ratio | Fold-Change |
|---|---|---|---|
| **VHL⁺ vs. VHL⁻ during normoxia** | | | |
| | KRT8 | 0.13 | –7.69 |
| | SOD2 | 0.25 | –4 |
| | ENO2 | 0.27 | –3.70 |
| | EZR | 0.31 | –3.23 |
| | SEPT11 | 0.35 | –2.86 |
| | PDCD6IP | 0.41 | –2.44 |
| | PPP1CC | 0.42 | –2.38 |
| | TPI1 | 0.45 | –2.22 |
| | GMPS | 0.46 | –2.17 |
| | ENO1 | 0.47 | –2.13 |
| | FAH | 0.47 | –2.13 |
| | AKR1B1 | 0.47 | –2.13 |
| | TPI1 | 0.48 | –2.08 |
| | PKM2 | 0.49 | –2.04 |
| | UQCRC1 | 2.00 | 2 |
| | PGAM1 | 2.02 | 2.02 |
| | G6PD | 2.02 | 2.02 |
| | HSPA4 | 2.16 | 2.16 |
| | SAHH | 2.75 | 2.75 |
| | ANXA4 | 2.78 | 2.78 |
| | VDAC1 | 2.89 | 2.89 |
| | PRDX3 | 2.92 | 2.92 |
| | APRT | 3.17 | 3.17 |
| | PRDX2 | 3.56 | 3.56 |
| | GSTP1 | 4.55 | 4.55 |
| | QPRT | 4.68 | 4.68 |
| | TXN | 6.08 | 6.08 |
| | UCHL1 | 6.28 | 6.28 |
| **VHL⁻ hypoxia vs. normoxia** | | | |
| | MSN | 2.0 | 2 |
| | XRCC5 | 2.1 | 2.1 |

*(Continued)*

| Setting | Gene Symbol | Ratio | Fold-Change |
|---|---|---|---|
| | ACO1 | 2.1 | 2.1 |
| | C22orf28 | 2.4 | 2.4 |
| | GANAB | 2.7 | 2.7 |
| | EIF3B | 2.7 | 2.7 |
| | EEF2 | 2.8 | 2.8 |
| | EIF5A | 3.7 | 3.7 |
| | VCL | 4.4 | 4.4 |
| **VHL$^+$ vs. VHL$^-$ during hypoxia** | | | |
| | CFL1 | 0.06 | –16.67 |
| | KRT19 | 0.22 | –4.55 |
| | PCNA | 0.32 | –3.13 |
| | TGM2 | 0.34 | –2.94 |
| | SOD2 | 0.34 | –2.94 |
| | FABP5 | 0.36 | –2.78 |
| | PSME1 | 0.38 | –2.63 |
| | KRT8 | 0.38 | –2.63 |
| | SERPINB9 | 0.42 | –2.38 |
| | CLR | 0.49 | –2.04 |
| | ACO1 | 0.49 | –2.04 |
| | MAPK1 | 2.01 | 2.01 |
| | VDAC1 | 2.03 | 2.03 |
| | GLRX3 | 2.05 | 2.05 |
| | WDR1 | 2.06 | 2.06 |
| | NAPA | 2.07 | 2.07 |
| | PPP2CA | 2.07 | 2.07 |
| | G6PD | 2.08 | 2.08 |
| | HSP90B1 | 2.09 | 2.09 |
| | ENO1 | 2.11 | 2.11 |
| | SDHA | 2.16 | 2.16 |
| | VDAC2 | 2.23 | 2.23 |
| | ALDOA | 2.30 | 2.3 |
| | ALDH9A1 | 2.36 | 2.36 |
| | EFHD2 | 2.39 | 2.39 |
| | SEPT8 | 2.40 | 2.4 |
| | PRDX1 | 2.66 | 2.66 |
| | ERP29 | 2.77 | 2.77 |

(*Continued*)

| Setting | Gene Symbol | Ratio | Fold-Change |
|---|---|---|---|
| | QPRT | 2.94 | 2.94 |
| | ECHS1 | 3.03 | 3.03 |
| | ANXA4 | 3.34 | 3.34 |
| | PRDX2 | 4.19 | 4.19 |
| | LPP | 4.50 | 4.5 |
| | RPS12 | 5.35 | 5.35 |
| | FHL2 | 8.78 | 8.78 |
| | UCHL1 | 9.67 | 9.67 |
| | GSTP1 | 12.3 | 12.3 |
| | PFD2 | 22.67 | 22.67 |
| | ADI1 | 29.66 | 29.66 |
| **VHL$^{+}$ hypoxia vs. normoxia** | | | |
| | FTL | 3.06 | 3.06 |
| | NDRG1 | 2.03 | 2.03 |

550 µg protein lysate isolated from VHL$^{-}$/VHL$^{+}$ 786–O cells incubated under normoxic or hypoxic (1 % $O_2$, 48 h) conditions respectively, were loaded onto IPG strips (pH 3–10, non–linear, Amersham Biosciences) followed by an isoelectric focusing and second–dimension SDS–PAGE separation (13 %) and staining with colloidal Coomassie as described (13). The gels were analyzed using the Delta2D software package (Decodon). Proteins found to be at least two–fold regulated (factor ≥ 2.0 or ≤ 0.50; $p \leq 0.05$) were subjected to mass spectrometric identification.

## Supplementary Table S6: Functional classification of differentially expressed metabolic proteins

| Metabolic function | VHL-dependent | hypoxia-dependent |
|---|---|---|
| Glycolysis | PKM2 ↓ ENO2 ↓TPI1 ↓ PGAM 1↑ | TPI1, ALDOA, ENO2 ↑ |
| Citrate cycle/mitochondrial respiratory chain | ACO1 ↓UQCRC1, ECHS1, SDHA ↑ | - |
| Fatty acid uptake | FABP5 ↓ | - |
| Energy metabolism | GMPS ↓ | GANAB ↑ |
| Crosslinking of proteins | TGM 2 ↓ ANXA4 ↑ | TGM2 ↑ |
| ROS degradation | PRDX2+3, GSTP1 ↑SOD2 ↓ | PRDX1+2, TXN ↑ |

Via 2DE-based proteome analysis followed by mass spectrometry identified proteins, as described in material and methods, were validated via qPCR and immunoblot analysis and classified in the different metabolic functions. ↑ up-regulated ↓ down-regulated.
